# Evaluation on Growth Performance, Nutrient Digestibility and Fecal Scores in Weaning Pigs Fed Wheat Bran- or Beet Pulp-Based Diets

**DOI:** 10.3390/ani16091326

**Published:** 2026-04-27

**Authors:** Zhenyu Ding, Jemin Ahn, In Ho Kim

**Affiliations:** 1 Department of Animal Biotechnology, Dankook University, Cheonan 31116, Republic of Korea; d72231721@dankook.ac.kr; 2 Smart Animal Bio Institute, Dankook University, Cheonan 31116, Republic of Korea; 3 College of Media, Tonghua Normal University, No. 950 Yucai, Dongchang, Tonghua 134002, China; 4 Daehan Feed Co., Ltd., 13, Bukseongpo-gil, Jung-gu, Incheon 22300, Republic of Korea; jeminahn@daehanfeed.co.kr

**Keywords:** growth performance, nutrient digestibility, insoluble fiber, soluble fiber, weaning pigs

## Abstract

This study investigated the effects of wheat bran and beet pulp as fiber-containing ingredients on growth performance, nutrient digestibility, and fecal score in weaned piglets. A total of 105 [(Landrace × Yorkshire) × Duroc] piglets, with an initial body weight of 6.65 ± 1.11 kg, were randomly allocated to three dietary treatments in a completely randomized design for 5 weeks. Each treatment has 7 replicates with 5 pigs per pen (two gilts and three barrows). The experimental period lasted 5 weeks and consisted of two feeding phases. The dietary treatments were as follows: CON, basal diet; TRT1, basal diet formulated with insoluble fiber (wheat bran); and TRT2, basal diet formulated with soluble fiber (beet pulp). Our results showed that pigs fed insoluble fiber (wheat bran-based diet) diet showed significantly increased average daily feed intake during weeks 3 to 5. However, neither insoluble nor soluble fiber affected nutrient digestibility or fecal score, indicating that the incorporation of these fiber sources at the tested levels did not impair digestive efficiency. In summary, the inclusion of insoluble fiber (wheat bran) in weaning diets enhance feed intake during the critical post-weaning adaptation period without compromising nutrient utilization or fecal consistency.

## Simple Summary

Weaning is a stressful period for piglets, often leading to reduced feed intake and poor growth. Finding simple and practical nutritional strategies to support piglets during this stage is important for animal health and farm productivity. Dietary fiber is widely used in pig diets, but different types of fiber may have different effects. In this study, we compared two common fiber sources, wheat bran and beet pulp, in diets for weaned piglets. We found that piglets fed wheat bran ate more feed during the later stage of the experiment, while beet pulp did not increase feed intake. However, neither fiber source improved growth rate or feed efficiency. Importantly, neither fiber sources did not negatively affected nutrient digestion or fecal consistency, indicating that they were well tolerated by the animals. These results suggest that wheat bran may help stimulate feed intake during the critical post-weaning period without harming digestion. This study provides practical information for designing piglet diets and supports the safe use of fiber ingredients in pig production systems.

## 1. Introduction

The weaning period represents a critical developmental window in piglets [1]. During this stage, piglets undergo abrupt separation from maternal milk, transition to solid feed, and exposure to environmental changes [2]. These combined stressors predispose them to “weaning stress syndrome,” which leads to reduced feed intake, impaired intestinal morphology, and compromised digestive and absorptive capacity [3]. Such disruptions not only hinder early growth performance but may also have lasting effects on health status and production efficiency [4,5]. Therefore, maintaining feed intake and supporting gastrointestinal functionality are primary nutritional objectives during this critical transition period. Dietary fiber has gained increasing attention as a functional component in swine nutrition due to its ability to regulate gastrointestinal physicochemical properties, modulate microbial composition, and influence digestive processes [6,7,8,9]. In weaned piglets, dietary fiber may alleviate weaning-associated stress by stabilizing the gut environment, promoting intestinal development, and enhancing microbial homeostasis, thereby contributing to improved adaptation to post-weaning diets. Based on solubility, dietary fiber is broadly classified into soluble and insoluble fractions, each exhibiting distinct physiological and functional characteristics [10,11]. Insoluble fiber (e.g., cellulose and lignin) primarily increases digesta bulk, stimulates intestinal motility, and accelerates digesta passage rate, which may enhance voluntary feed intake and feeding behavior [12]. In contrast, soluble fiber (e.g., pectin and β-glucan) increases digesta viscosity, delays gastric emptying, and serves as a fermentable substrate for beneficial microbiota such as *Lactobacillus* [13,14,15]. Despite these recognized functional differences, most previous studies have primarily evaluated the effects of individual fiber types in weaned piglets [16,17,18]. Soluble fiber has been reported to improve intestinal barrier integrity and gut health [8,19]), whereas insoluble fiber may enhance feed intake by stimulating gastrointestinal motility and reducing digesta retention time [6,20].

However, the physiological effects of dietary fiber are not always consistent across studies, as the response may vary depending on fiber source, inclusion level, and physicochemical properties such as solubility, viscosity, and fermentability. Soluble fiber has been reported to improve intestinal barrier function and gut health, whereas insoluble fiber may enhance feed intake by influencing digesta bulk and passage rate [6,10,18]. In addition, while improving feed intake is considered a key nutritional strategy to alleviate post-weaning growth lag, the relationship between increased feed intake and growth performance under different fiber types remains unclear, particularly in the context of potential energy dilution and altered nutrient utilization [21,22,23]. Therefore, a systematic evaluation of fiber type on both intake behavior and nutrient utilization is warranted. In the present study, wheat bran and beet pulp were selected as representative fiber-containing ingredients. Wheat bran is widely used in swine diets and is characterized by a high content of insoluble non-starch polysaccharides, whereas beet pulp is considered a highly fermentable fiber source rich in pectic substances [6,13,20]. Based on these characteristics, this study aimed to evaluate the effects of wheat bran- and beet pulp-based diets on growth performance, nutrient digestibility, and fecal consistency in weaned piglets. By directly comparing these two functional fiber types, this study will provide practical knowledge on optimizing fiber utilization in weaning diets.

## 2. Materials and Methods

### 2.1. Ethical Statement

All animal experiments complied with established ethical guidelines and were approved by the Institutional Animal Care and Use Committee of Dankook University, Republic of Korea (Approval No. DK-2-2419; 20 August 2024).

### 2.2. Animals, Husbandry Management and Diets

A total of 105 [(Landrace × Yorkshire) × Duroc] weaned piglets, with an average initial body weight of 6.65 ± 1.11 kg, were used in this study. The piglets were randomly assigned to three dietary treatments in a completely randomized design, with 7 replicate pens per treatment and 5 pigs per pen. Each pen was considered the experimental unit. The pigs were housed in an environmentally controlled nursery room maintained at approximately 30 °C during the first week and gradually reduced thereafter, and each pen provided approximately 1.8 m^2^ of floor space (approximately 1.5 m × 1.2 m per pen), equipped with a feeder and an automatic nipple drinker.

The experiment was conducted over 5 weeks and consisted of two dietary phases (phase 1: week 1–3; phase 2: week 3–5). The dietary treatments were as follows: CON, basal diet; TRT1, basal diet formulated with insoluble fiber (wheat bran); and TRT2, basal diet formulated with soluble fiber (beet pulp). The basal diet was formulated to meet or exceed the nutrient requirements of weaned piglets according to the National Research Council [24]. The basal diet (CON) was formulated as a standard weaning diet and served as a reference control without additional fiber-containing ingredients. The dietary treatments (TRT1 and TRT2) were formulated by incorporating wheat bran and beet pulp, respectively, as alternative fiber-containing ingredients. Although wheat bran and beet pulp are commonly regarded as representative sources of predominantly insoluble and soluble fiber, respectively, the present study did not include direct analytical measurements of soluble and insoluble dietary fiber fractions. Therefore, the comparison in this study should be interpreted primarily as an evaluation of different fiber-containing ingredients rather than a strictly controlled comparison of fiber solubility fractions. The ingredient composition and calculated value are presented in Table 1 and Table 2. The Wheat bran and beet pulp were commercially purchased from Daehan Feed Co., Ltd. (Incheon, Republic of Korea). and added into basal diet. All piglets had ad libitum access to feed and water throughout the experimental period via an automatic watering system.

### 2.3. Sampling and Analysis

Body weight (BW) of individual piglets was recorded at the end of weeks 1, 3, and 5. Individual values within each pen were averaged to obtain pen-based means, and the pen was considered the experimental unit for statistical analysis. Feed intake was recorded on a pen basis and used to calculate average daily gain (ADG), average daily feed intake (ADFI), and feed conversion ratio (FCR).

Apparent total tract digestibility (ATTD) of dry matter (DM), nitrogen (N), and energy (E) was determined using chromium oxide (Cr_2_O_3_) as an indigestible marker at an inclusion level of 0.5% in the diet. A 5-day adaptation period was allowed for marker equilibration, followed by fecal sample collection. Fecal samples were collected by rectal massage from each pen once daily during the final 3 consecutive days of each sampling period and pooled within pen. All samples were immediately stored at −20 °C until analysis. Prior to chemical analysis, fecal samples were dried at 60 °C for 72 h and ground using a Wiley mill (Model 4, Thomas Scientific, Swedesboro, NJ, USA). Subsamples were further dried at 105 °C for 24 h to determine dry matter content, and all samples were ground to pass through a 1 mm screen. The concentrations of DM, N, and energy in feed and fecal samples were analyzed according to standard procedures described by AOAC (2003) [25]. Fecal consistency was visually assessed using a previously described 5-point scoring system (1 = hard, dry pellets; 2 = firm, well-formed feces; 3 = soft, formed, and moist feces; 4 = soft, unformed feces; and 5 = watery, liquid feces) according to a previously described method [26].

### 2.4. Statistical Analysis

All data were analyzed using the General Linear Model (GLM) procedure of SAS (Version 9.4, SAS Institute Inc., Cary, NC, USA). Pen was used as an experimental unit. Data was analyzed in a completely randomized design with dietary treatment as the fixed effect. Differences among treatment means were determined using Duncan’s multiple range test. Statistical significance was declared at *p* < 0.05, and trends were discussed at 0.05 ≤ *p* < 0.10. Results are presented as means along with the pooled standard error of the mean (SEM).

## 3. Results

The effects of dietary insoluble and soluble fiber on growth performance, nutrient digestibility, and fecal score are presented in Table 3, Table 4 and Table 5. Piglets fed the insoluble fiber (wheat bran)-based diet showed higher ADFI during weeks 3 to 5 compared with the control and soluble fiber diets, whereas no significant differences were observed in ADG or FCR. No significant effects were found on ATTD of dry matter, nitrogen, or energy, and fecal score was also unaffected by dietary treatments.

## 4. Discussion

The results showed that pigs fed the wheat bran diet had higher average daily feed intake during weeks 3 to 5 compared with the other treatments. This is consistent with previous findings indicating that wheat bran, as a typical source of insoluble fiber, can increase voluntary feed intake by stimulating gastrointestinal motility and accelerating the digesta passage rate [27]. As a structural fiber, wheat bran increases digesta bulk [28], thereby accelerating gastric emptying [29] and attenuating the inhibitory effects of satiety signals on central feed intake regulation [30].

In contrast, the soluble fiber diet based on beet pulp did not exhibit a similar stimulatory effect on ADFI. Beet pulp is rich in pectic polysaccharides and is highly fermentable in the hindgut, which may influence digestive processes and fecal characteristics, particularly acetate and butyrate [31]. Additionally, the high water-holding capacity and moderate viscosity of beet pulp may delay gastric emptying and prolong digesta retention time, which could further enhance satiety. Despite the significant increase in ADFI in the wheat bran group, no improvements were observed in ADG or FCR. This can be primarily attributed to the energy dilution effect associated with the addition of fiber. The substitution of energy-dense ingredients with wheat bran reduces dietary energy density [32], such that increased feed intake does not necessarily result in increased net energy intake. Furthermore, the accelerated passage rate may reduce the contact time between nutrients and digestive enzymes, potentially limiting nutrient digestion efficiency. The lack of significant improvements in growth performance may also be related to the relatively short experimental duration, as the effects of dietary fiber on gut function are often cumulative and may become more evident over longer feeding periods or at later production stages.

These findings indicate that the inclusion levels of wheat bran and beet pulp used in the present study were well tolerated by weaned piglets, as no negative effects were observed on nutrient digestibility. Although high levels of dietary fiber have been reported to reduce nutrient digestibility due to increased digesta passage rate and dilution effects [33,34], moderate inclusion levels may minimize such negative impacts. Previous studies have also shown that the inclusion of wheat bran or sugar beet pulp at appropriate levels does not markedly impair apparent nutrient digestibility in pigs [35,36]. Under the present experimental conditions, the inclusion of these fiber sources as basal dietary components did not adversely affect nutrient utilization or growth performance. Future studies, including quantitative analysis of soluble and insoluble fiber fractions, are warranted to further validate these findings.

Fecal score was included in the present study as an important indicator of post-weaning adaptation and intestinal health in piglets. In weaned pigs, fecal consistency is widely used as a practical and non-invasive measure to assess intestinal function and diarrhea incidence, which are closely associated with overall health status and adaptation to dietary changes [6,13,18]. Increased fecal scores are generally indicative of impaired intestinal function and a higher risk of post-weaning diarrhea, whereas stable fecal scores suggest better adaptation and physiological stability. In the present study, no significant differences in fecal score were observed among treatments throughout the experimental period, indicating that the inclusion of wheat bran or beet pulp did not adversely affect fecal consistency. This finding suggests that the inclusion levels of both fiber sources were within the tolerance range of weaned piglets. Previous studies have reported that the effects of dietary fiber on fecal characteristics are highly dependent on fiber source and inclusion level [16,20]. Under the present experimental conditions, the absence of treatment effects may be attributed to the moderate inclusion levels and the lack of a severe intestinal challenge during the study period, which may have limited the observable differences in fecal score [35]. Overall, these results indicate that the inclusion of wheat bran or beet pulp at the tested levels was well tolerated without negatively affecting fecal consistency in weaned piglets.

## 5. Conclusions

In conclusion, the inclusion of wheat bran or beet pulp as dietary components did not significantly affect growth performance or nutrient digestibility but influenced feed intake patterns. Wheat bran increased average daily feed intake, whereas beet pulp maintained similar digestibility and fecal consistency compared with the control diet. These findings indicate that both fiber sources were well tolerated under the present experimental conditions.

## Figures and Tables

**Table 1:** Composition of weaning pig diets. (Phase 1).

Item	CON	TRT1 Insoluble Fiber (Wheat Bran)	TRT2 Soluble Fiber (Beet Pulp)
Ingredients (%)			
Corn	51.12	50.54	50.83
Soybean meal	21.16	19.46	20.09
Wheat	10.00	10.00	10.00
Wheat bran	-	2.50	-
Beet pulp	-	-	1.50
Fish meal	5.00	5.00	5.00
Blood plasma protein	1.67	1.67	1.67
Soy oil	3.89	3.70	3.78
Lactose	3.62	3.62	3.62
MCP	0.41	0.33	0.37
Limestone	0.63	0.66	0.62
Salt	0.20	0.20	0.20
Lysine (78%)	0.61	0.62	0.62
Methionine (98%)	0.35	0.35	0.35
Threonine (99%)	0.35	0.35	0.35
Tryptophan (99%)	0.11	0.11	0.11
Valine (98%)	0.21	0.22	0.22
Mineral mix ^1^	0.20	0.20	0.20
Vitamin mix ^2^	0.20	0.20	0.20
ZnO (80%)	0.18	0.18	0.18
Choline (25%)	0.10	0.10	0.10
Total	100.00	100.00	100.00
Calculated value			
Crude protein, %	18.30	18.30	18.30
Net Energy, kcal/kg	2700	2700	2700
Calcium, %	0.55	0.55	0.55
Phosphorus, %	0.50	0.50	0.50
SID Lysine, %	1.35	1.35	1.35
SID Methionine, %	0.61	0.61	0.61
SID Threonine	0.92	0.92	0.92
SID Tryptophan	0.27	0.27	0.27
SID Valine	0.92	0.92	0.92
Crude Fat, %	8.36	8.26	8.29
Crude Fiber, %	3.16	2.55	2.71
Crude Ash, %	4.91	4.80	4.85
Lactose, %	10.00	10.00	10.00

^1^ Provided per kg diet: Fe, 100 mg as ferrous sulfate; Cu, 17 mg as copper sulfate; Mn, 17 mg as manganese oxide; I, 0.5 mg as potassium iodide; and Se, 0.3 mg as sodium selenite. ^2^ Provided per kilograms of diet: vitamin A, 10,800 IU; vitamin D3, 4000 IU; vitamin E, 40 IU; vitamin K3, 4 mg; vitamin B1, 6 mg; vitamin B2, 12 mg; vitamin B6, 6 mg; vitamin B12, 0.05 mg; biotin, 0.2 mg; folic acid, 2 mg; niacin, 50 mg; D-calcium pantothenate, 25 mg.

**Table 2:** Composition of weaning pig diets. (Phase 2).

Item	CON	TRT1 Insoluble Fiber (Wheat Bran)	TRT2 Soluble Fiber (Beet Pulp)
Ingredients (%)			
Corn	47.91	47.61	48.12
Soybean meal	22.74	21.90	21.11
Wheat	15.00	15.00	15.00
Wheat bran	-	1.25	-
Beet pulp	-	-	1.50
Fish meal	5.00	5.00	5.00
Soy oil	4.08	3.96	3.97
Lactose	1.67	1.67	1.67
MCP	0.62	0.64	0.65
Limestone	0.70	0.70	0.69
Salt	0.20	0.20	0.20
Lysine (78%)	0.56	0.57	0.56
Methionine (98%)	0.29	0.29	0.29
Threonine (99%)	0.31	0.31	0.31
Tryptophan (99%)	0.08	0.08	0.09
Valine (98%)	0.15	0.15	0.15
Mineral mix ^1^	0.20	0.20	0.20
Vitamin mix ^2^	0.20	0.20	0.20
ZnO (80%)	0.18	0.18	0.18
Choline (25%)	0.10	0.10	0.10
Total	100.00	100.00	100.00
Calculated value			
Crude protein, %	18.30	18.30	18.30
Net Energy, kcal/kg	2700	2700	2700
Calcium, %	0.55	0.55	0.55
Phosphorus, %	0.52	0.52	0.52
SID Lysine, %	1.25	1.25	1.25
SID Methionine, %	0.56	0.56	0.56
SID Threonine	0.85	0.85	0.85
SID Tryptophan	0.25	0.25	0.25
SID Valine	0.85	0.85	0.85
Crude Fat, %	8.89	8.85	8.83
Crude Fiber, %	3.13	2.83	2.97
Crude Ash, %	4.89	4.82	4.85
Lactose, %	1.64	1.64	1.64

^1^ Provided per kg diet: Fe, 100 mg as ferrous sulfate; Cu, 17 mg as copper sulfate; Mn, 17 mg as manganese oxide; I, 0.5 mg as potassium iodide; and Se, 0.3 mg as sodium selenite. ^2^ Provided per kilograms of diet: vitamin A, 10,800 IU; vitamin D3, 4000 IU; vitamin E, 40 IU; vitamin K3, 4 mg; vita-min B1, 6 mg; vitamin B2, 12 mg; vitamin B6, 6 mg; vitamin B12, 0.05 mg; biotin, 0.2 mg; folic acid, 2 mg; niacin, 50 mg; D-calcium pantothenate, 25 mg.

**Table 3:** The effect of dietary insoluble and soluble fiber on growth performance in weaned piglets ^1^.

Item	CON	TRT1 Insoluble Fiber (Wheat Bran)	TRT2 Soluble Fiber (Beet Pulp)	SEM ^2^	*p*-Value
Body weight, kg					
Initial	6.66	6.65	6.65	0.004	0.252
Week 1	8.33	8.34	8.31	0.02	0.655
Week 3	13.55	13.67	13.46	0.10	0.383
Week 5	20.65	20.91	20.46	0.21	0.344
Initial–Week 1					
ADG, g	239	242	237	4	0.662
ADFI, g	276	282	272	4	0.847
FCR	1.156	1.173	1.148	0.006	0.658
Week 1–Week 3					
ADG, g	373	380	368	7	0.451
ADFI, g	484	499	479	9	0.090
FCR	1.297	1.314	1.302	0.019	0.854
Week 3–Week 5					
ADG, g	507	518	501	10	0.477
ADFI, g	788 ^b^	810 ^a^	774 ^b^	15	0.044
FCR	1.552	1.578	1.550	0.028	0.698
Overall					
ADG, g	400	408	395	6	0.357
ADFI, g	564	580	555	8	0.082
FCR	1.407	1.390	1.414	0.017	0.621

^1^ Abbreviation: CON: basal diet; TRT1: Insoluble fiber (Wheat bran); TRT2: Soluble fiber (Beet pulp). ^2^ Standard error of means. ^a,b^ Means in the same row with different superscripts differ significantly (*p* < 0.05).

**Table 4:** The effect of dietary insoluble and soluble fiber on nutrient digestibility in weaned piglets ^1^.

Item	CON	TRT1 Insoluble Fiber (Wheat Bran)	TRT2 Soluble Fiber (Beet Pulp)	SEM ^2^	*p*-Value
Week 1					
Dry matter	90.45	90.80	90.31	0.62	0.945
Nitrogen	82.75	82.99	82.64	0.29	0.808
Energy	88.03	88.39	87.88	0.42	0.983
Week 3					
Dry matter	88.28	88.75	88.08	0.19	0.597
Nitrogen	80.74	81.10	80.53	0.44	0.802
Energy	84.98	85.29	84.49	0.64	0.857
Week 5					
Dry matter	86.91	87.36	86.52	0.57	0.403
Nitrogen	78.36	78.85	78.02	0.29	0.224
Energy	81.40	82.51	81.28	0.51	0.187

^1^ Abbreviation: CON: basal diet TRT1: Insoluble fiber (Wheat bran); TRT2: Soluble fiber (Beet pulp). ^2^ Standard error of means.

**Table 5:** The effect of dietary insoluble and soluble fiber on fecal score in weaned piglets ^1^.

Item	CON	TRT1 Insoluble Fiber (Wheat Bran)	TRT2 Soluble Fiber (Beet Pulp)	SEM ^2^	*p*-Value
Fecal score ^3^					
Initial	3.61	3.59	3.63	0.06	0.886
Week 1	3.58	3.54	3.56	0.05	0.844
Week 3	3.49	3.44	3.47	0.06	0.829
Week 5	3.34	3.26	3.32	0.04	0.305

^1^ Abbreviation: CON: basal diet; TRT1: Insoluble fiber (Wheat bran); TRT2: Soluble fiber (Beet pulp). ^2^ Standard error of means. ^3^ Fecal score = 1 hard, dry pellet; 2 firm, formed stool; 3 soft, moist stool that retains shape; 4 soft, unformed stool that assumes shape of container; 5 watery liquid that can be poured.

## Data Availability

Data are available on request due to privacy.

## Abbreviations

The following abbreviations are used in this manuscript:

BW	Body weight
ADG	Average daily gain
ADFI	Average daily feed intake
FCR	Feed conversion ratio
ATTD	Apparent total tract digestibility
DM	Dry matter
N	Nitrogen
E	Energy
GLM	General linear model
SEM	Standard error of mean
